# Detection of intracranial aneurysms using deep learning-based CAD system: usefulness of the scores of CNN’s final layer for distinguishing between aneurysm and infundibular dilatation

**DOI:** 10.1007/s11604-022-01341-7

**Published:** 2022-09-29

**Authors:** Makiko Ishihara, Masato Shiiba, Hirotaka Maruno, Masayuki Kato, Yuki Ohmoto-Sekine, Choppin Antoine, Yasuyoshi Ouchi

**Affiliations:** 1 Department of Diagnostic Imaging Center, Toranomon Hospital, 1-8-1 Akasaka Intercity AIR 5F, Akasaka, Minato-ku, Tokyo, 107-0052 Japan; 2 Department of Radiology, Toranomon Hospital, 2-2-2 toranomon, Minato-ku, Tokyo, 105-8470 Japan; 3 Department of Health Management Center, Toranomon Hospital, 1-8-1 Akasaka Intercity AIR 5F, Akasaka, Minato-ku, Tokyo, 107-0052 Japan; 4 LPIXEL Inc, 1-6-1 Ohtemachi, Chiyoda-ku, Tokyo, 100-0004 Japan; 5 Department of Geriatric Medicine, Toranomon Hospital, 2-2-2 toranomon, Minato-ku, Tokyo, 105-8470 Japan

**Keywords:** Unruptured aneurysm, Infundibular dilatation, Deep learning-based CAD, Convolutional neural network’s final layer

## Abstract

**Purpose:**

We evaluated the diagnostic performance of a clinically available deep learning-based computer-assisted diagnosis software for detecting unruptured aneurysms (UANs) using magnetic resonance angiography and assessed the functionality of the convolutional neural network (CNN) final layer score for distinguishing between UAN and infundibular dilatation (ID).

**Materials and methods:**

EIRL brain aneurysm (EIRL_BA) was used in this study. The subjects were 117 UAN and/or ID cases including 100 UAN lesions (average sizes of 2.56 ± 1.45 mm) and 40 ID lesions (average sizes of 1.75 ± 0.41 mm) in any of internal carotid artery, middle cerebral artery, and anterior communicating artery, and 123 normal controls. The sensitivity, specificity, and accuracy of EIRL_BA were determined for UAN and ID or UAN only. Furthermore, the relationship between the lesion category and score was examined using a linear regression analysis model, and the receiver operating characteristic (ROC) analysis was used to assess whether the scores represent UAN-like characteristics.

**Results:**

EIRL_BA showed a total of 203 candidates (an average of 1.73/case) in UAN and/or ID cases and 98 candidates (an average of 0.80/case) in normal controls. For diagnosing either UAN/ID, EIRL_BA showed an overall sensitivity of 80%, specificity of 84.2%, and accuracy of 83.7%, resulting in the positive likelihood ratio of 5.0. For diagnosing UAN only, the overall sensitivity of 89.0, specificity of 82.6%, and accuracy of 83.2% resulting in the positive likelihood ratio of 5.1. In a linear regression analysis, the scores significantly increased in the candidates’ first and second ranks in UAN (*p* < 0.05) but not in ID. An ROC analysis using the score for diagnosing UAN showed an area under the curve of 0.836.

**Conclusion:**

EIRL_BA is applicable for detecting small UAN, and the CNN’s final layer scores may be an effective index for discriminating UAN and ID and representing the likelihood of UAN.

## Introduction

In Japan, “Brain dock” started in 1988 and has been practiced in many medical institutions since. It is a voluntary examination using magnetic resonance imaging and magnetic resonance angiography (MRA) to inspect the brain exhaustively, including the investigation whether unruptured aneurysms (UAN) exist or not [1]. One of the reasons for the popularity of “brain dock” is because the incidence of a rupture of UAN that causes subarachnoid hemorrhage and frequently leads to fatal outcomes [2] is 2.8 times higher in Japanese than that in Westerners [3]. Therefore, accurate diagnosis of UAN by MRA is a crucial issue for diagnostic physicians.

However, the sensitivity of 1.5 T MRA to detect UAN is as low as 60–79% of digital subtraction angiography, which is the gold standard, with particularly low sensitivity in the regions of the internal carotid artery with less than 3 mm and the anterior cerebral artery [4]. Moreover, differentiation of infundibular dilatations (ID), initially reported as funnel-shaped dilatations at the origin of the posterior communicating artery [5, 6] is sometimes difficult because of their morphological resemblance, despite the importance of accurate diagnosis due to the distinctive follow-up strategies [7, 8].

In this situation, the development of computer-assisted diagnosis (CAD) for detecting intracranial UAN on MRA has been attempted [9–13], and a deep learning-based CAD system has been recently developed [14–19]. EIRL brain aneurysm^®^ (EIRL_BA), approved for pharmaceutical use in 2019, is one of the deep learning-based CAD systems using a convolutional neural network (CNN). It is based on the algorithm where the shape and curvature of the vascular signal on the original MRA images are quantified for determining aneurysmal candidates [10]. Therefore, the scores of the CNN’s final layer, that is, the final results of the algorithm, are derived from the index of the morphological characteristics of the disease and are supposed to be able to use as the confidence level of CAD [20]. However, the validity of these scores has not been fully examined.

This study aims to evaluate the diagnostic performance of a clinically available CAD software for detecting UAN with a special interest in distinguishing UAN and ID.

## Materials and methods

### Ethics statement

This retrospective study was approved by the ethics committee of our hospital (No.1825). Written informed consent was waived due to the retrospective nature of the study.

### Subjects

The subjects included men/women who underwent “brain dock” in our hospital from October 2017 to March 2019. In this retrospective study, subjects were picked up from the “brain dock” examinees’ database which linked to a simple diagnostic name. First, 120 cases with a diagnostic name “aneurysm” or “infundibulum” and 120 cases with age-matched “normal” cases were extracted. However, there was no record of the location or size of the lesion, and a sole reader diagnosed some cases. Therefore, two board-certified general radiologists (M.I., with 32 years of experience, M.S., with 20 years of experience, respectively) reviewed the images again. When the opinions of the two radiologists differed, the final diagnosis was determined by a consensus. As a result of a review by two radiologists, among the 120 cases that had been labeled with the diagnostic name “normal,” UAN or ID was found in 29 cases, and among the 120 cases with a diagnosis of “UAN/ID,” 32 cases did not have lesions in the target vascular regions. Therefore, the subject sample was ultimately divided into two groups: the group with cerebral aneurysm or ID in the left and/or right internal carotid arteries, the middle cerebral artery (M1–M2), or the anterior communicating artery, consisting of 117 cases with 140 lesions; and the normal group, consisting of 123 cases.

Finally, 240 cases were divided into 117 cases with 140 lesions having UAN and/or ID in either the internal carotid artery (ICA), middle cerebral artery (MCA; M1–M2), or anterior communicating artery (A-com) (= UAN/ID group), and 123 normal cases having no lesions in the three vessels (= normal control group).

The diagnostic criterion for UAN was a dome-shaped ridge protruding from the vessel in the artery wall or vascular branch which was observable by maximum intensity projection (MIP) images of at least two of three directions. The diagnostic criterion for ID was a funnel-shaped structure of < 3 mm at the bifurcation, without an aneurysmal neck, with peripheral blood vessels observed at its tip according to the definition [8]. However, we diagnosed ID not limited to the ICA, because it was often observed in other vascular areas. The source images of MRA were evaluated if necessary.

### MRA image acquisition in the “Brain Dock”

All the MRA examinations were performed on 1.5 T equipment (Philips Ingenia, Philips Medical Systems, Netherlands) with a dStream Headspine coil. 3D Time-of-flight MRA data were obtained from the cranial base to the main trunk of MCA under the following parameters: repetition time/echo time 24/6.91 ms, flip angle 18°, a field of view 200 × 200 mm, acquisition matrix 352 × 223, and slice thickness 0.5 mm. MIP images were reconstructed from the original MRA images with every 15° of transverse, left and right, and coronal direction.

### Analysis of MRA images by EIRL_BA

A clinically available deep learning-based CAD software (EIRL brain aneurysm (EIRL_BA), version 1.7, LPIXEL Inc., Tokyo, Japan) was used to evaluate UAN/ID. In the current version, the algorithm consists of a two-stage CNN architecture.

**Fig. 1:**
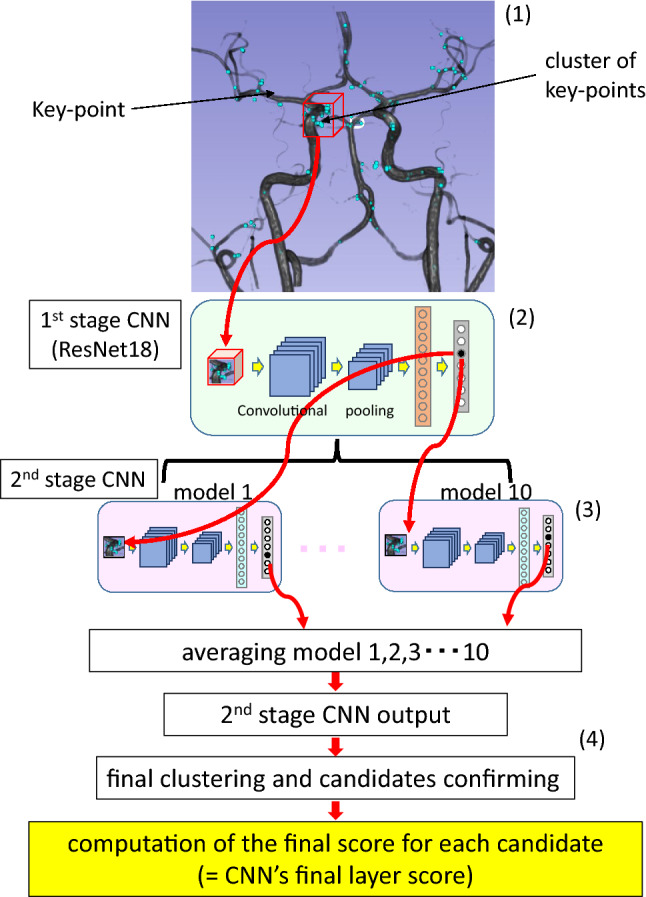
Overview of the algorithm and two-stage CNN architecture in EIRL brain aneurysm^®^ to detect unruptured aneurysm. 1 Each bright blue dot represents a single “key-point” (that is, a point for which the principal curvature and shape index of the MRA data are above a specific threshold). A 3D patch represented by a red wireframe box is extracted around each key-point. 2 Extracted 3D patches are fed into the first stage CNN (ResNet18). 3 The output of the first stage CNN yields a value between 0 and 1 corresponding to each key-point, and 3D patches around key-points with the first CNN’s output value above 0.55 are fed into the second-stage CNN (an ensemble of 10 CNNs). 4 Key-points (for which the average of the output of the 10 CNNs is above 0.27) are again clustered to compute the CNN’s final layer score of the candidate. *CNN* convolution neural network, *MRA* magnetic resonance angiography

Analysis of MRA images by EIRL_BA was as follows (Fig. 1). First, the signal of the original MRA images is normalized and vessel extraction is performed. Next, for each pixel, the principal curvatures and the shape index are computed based on the curvature-based enhanced display system for detecting lesions in MRA [10]. The pixels for which any of these values above a certain threshold are selected as “key-points.” A 3D patch surrounding each key-point is then extracted and input into the first CNN (ResNet18). The output of the CNN is a continuous number between 0.0 and 1.0. In the first stage of CNN, key-points with an output below 0.55 are excluded, and the remaining key-points are clustered. The threshold of the first level CNN was kept to the original value validated by Ueda et al. [17]. A 3D patch around each key-point of the remaining clusters is extracted and input into the second stage of CNN (an ensemble of ten CNNs with a sigmoid activation function). The threshold of the second-stage CNN was tuned to 0.27 to reduce the number of false positives, while keeping the sensitivity the same as in the previous software version. Key-points below 0.27 were excluded. Many key-points still remained, and the remaining key-points were again clustered to confirm the scores of CNN’s final layer of the candidate and their locations were the centroid of key-points in the cluster. The clusters with the highest score are considered most similar to UAN. Up to four lesion candidates are determined based on the scores of CNN’s final layer in descending order. No candidate is displayed if no cluster has a score above the exclusion threshold.

EIRL-BA was connected to the PACS (picture archiving and communication system) in our hospital. The original MRA images were analyzed using the above procedure to determine the candidates’ presence and rank, and the CNN’s final layer score in the following vascular areas, including left and right ICA, left and right MCA, and A-com.

### Statistical analysis

Candidates detected by EIRL_BA were assigned to one of the two lesion types (UAN and ID), which derived from the diagnosis of two board-certified radiologists (M.I., with 32 years of experience, M.S., with 20 years of experience, respectively) as the gold standard, or normal (false positive). Then, the positive diagnostic rate of EIRL_BA was calculated using a quadrant table for each vascular region. Also, since a candidate is depicted as a circle of 10 mm in diameter, EIRL-BA detection was considered accurate if the lesion diagnosed by the radiologist was within or on the circle. However, since scores were represented only in the MRA original images, in some cases, it was hard to tell whether the candidate points matched the lesion diagnosed by the radiologist, and if they were not far apart, the lesion was diagnosed as a match. Also, the candidate points of EIRL_BA that matched the radiologists’ diagnosis, the false positive candidate points in the regional vessels, and the candidate points in the regional vessels in the normal group (all false positives) were then tabulated for each candidate point rank, and the median score was determined. Distribution of the numbers and median scores of the candidates in UAN, ID lesions, and normal were aggregated for each candidate level.

The diagnostic ability of EIRL_BA was shown in a quadrant table for each vascular area. When multiple candidates were shown in one vascular area, it was considered accurate if the true candidate was included. Then, a linear regression analysis model was used to predict scores based on the lesion types in each candidate’s rank and see if UAN or ID significantly increased scores when normal scores were used as a reference. The scores were not compared between the groups, because they did not show Gaussian distribution. Candidates 3 and 4 were calculated together because candidate 4, the lowest score among the candidates, had only one UAN, no ID, and eight false positive lesions.

Finally, a receiver operating characteristic (ROC) analysis was performed with the scores of the candidates diagnosed as UAN to assess whether the score of our data represents the likelihood of UAN accurately. Statistical analyses were performed using Excel (Microsoft Corporation, Redmond, WA, USA) and Stata 17 (StataCorp LLC, College Station, TX, USA). A *p* value of < 0.05 was considered statistically significant.

## Results

The subject characteristics of the UAN/ID and normal control groups are summarized in Table 1. In the UAN/ID group of 117 cases, a total of 140 lesions, including 96 cases of single lesions, 19 cases of double lesions, and 2 cases of triple lesions, were observed. In our cases, multiple lesions were not observed in a single vascular area. The average UAN (*n* = 100) size was 2.56 ± 1.45 mm, and 20/100 lesions were 3 mm or more. ID (*n* = 40) average size was 1.75 ± 0.41 mm, and all lesions were less than 3 mm by definition. The lesions were mostly located in ICA (*n* = 104), followed by A-com (*n* = 23) and MCA (*n* = 13).

**Table 1:** Characteristics of the subjects of UAN/ID and normal control groups

	Normal	UAN/ID	UAN	ID
Sex				
Male	79	71		
Female	44	46		
Age				
Younger than 50 y/o	26	16		
50–69 y/o	81	83		
70 y/o and older	16	18		
Multiplicity				
Single		96	73	23
Double		19	24	14
Triple		2	3	3
UAN/ID				
Size				
Less than 2 mm		56	28	28
2–2.9 mm		64	52	12
3–3.9 mm		10	10	0
4–4.9 mm		5	5	
More than 5 mm		5	5	0
Location				
Rt.ICA		64	49	15
Lt.ICA		40	28	12
Rt.MCA		7	5	2
Lt.MCA		6	3	3
A-com		23	15	8

*UAN* unruptured aneurysm, *ID* infundibular dilatation, *y/o* years old, *Rt* right, Lt left, *ICA* internal carotid artery, *MCA* middle cerebral artery, *A-com* anterior communicating artery

Table 2 shows the distribution of the candidates detected by EIRL_BA. EIRL_BA detected 301 candidates in 240 cases, including 123 cases of normal control. In the UAN/ID group, there were 203 candidates in 185/585 vascular areas, resulting in an average of 1.73 candidates per case. In the normal group, there were 98 candidates in 95/615 vascular areas, resulting in an average of 0.80 candidates per case. The median score was highest in UAN candidate 1, and the median scores of UAN were higher than those of ID or false positive lesions.

**Table 2:** The number of candidates and the median scores of CNN’s final layer for each group

	UAN/ID group			Normal control group
	UAN lesions	ID lesions	false positive lesions	false positive lesions
Candidate 1	69	12	19	62
median scores	0.995	0.759	0.893	0.636
Candidate 2	17	6	38	25
median scores	0.777	0.596	0.641	0.431
Candidate 3	2	5	26	11
median scores	0.638	0.459	0.429	0.338
Candidate 4	1	0	8	0
Median scores	0.535		0.407	

*CNN* convolutional neural network, *UAN* unruptured aneurysm, *ID* infundibular dilatation

The diagnostic accuracy of EIRL_BA in each vascular area is shown in Table 3. The sensitivity and specificity of EIRL_BA for detecting either UAN or ID lesions were 81.7 and 72.9% in ICA, 76.9 and 95.1% in MCA, and 73.9 and 80.2% in A-com, respectively. In total, sensitivity, specificity, and the accuracy were 80.0, 84.2, and 83.7%, resulting in the positive likelihood ratio of 5.0. Then, we evaluated the performance of EIRL_BA for diagnosing UAN only by excluding ID lesions from the true positive lesions. The sensitivity and the specificity for detecting UAN alone were 89.6 and 70.7% in ICA, 100 and 94.7% in MCA, and 80.0 and 78.7% in A-com, respectively. In total, the sensitivity and the specificity, and the accuracy for detecting UAN alone were 89.0 and 82.6%, and 83.2%, resulting in a positive likelihood ratio of 5.1.

**Table 3:** Summary of the diagnostic accuracy of the 3 vessel lesions

UAN/ID	ICA		MCA		A-com		Total	
sensitivity	85/104	81.7%	10/13	76.9%	17/23	73.9%	112/140	80.0%
specificity	274/376	72.9%	444/467	95.1%	174/217	80.2%	892/1060	84.2%
accuracy	359/480	74.8%	454/480	94.6%	191/240	79.6%	1004/1200	83.7%
LR( +)	3.0		15.6		3.7		5.0	

UAN	ICA		MCA		A-com		total	
sensitivity	69/77	89.6%	8/8	100%	12/15	80.0%	89/100	89.0%
specificity	285/403	70.7%	447/472	94.7%	177/225	78.7%	909/1100	82.6%
accuracy	354/480	73.8%	455/480	94.8%	189/240	78.8%	998/1200	83.2%
LR( +)	3.1		18.9		3.8		5.1	

*UAN* unruptured aneurysm, *ID* infundibular dilatation, *ICA* internal carotid artery, *MCA* middle cerebral artery, *A-com* anterior communicating artery, *LR (* +*)* positive likelihood ratio

**Fig. 2:**
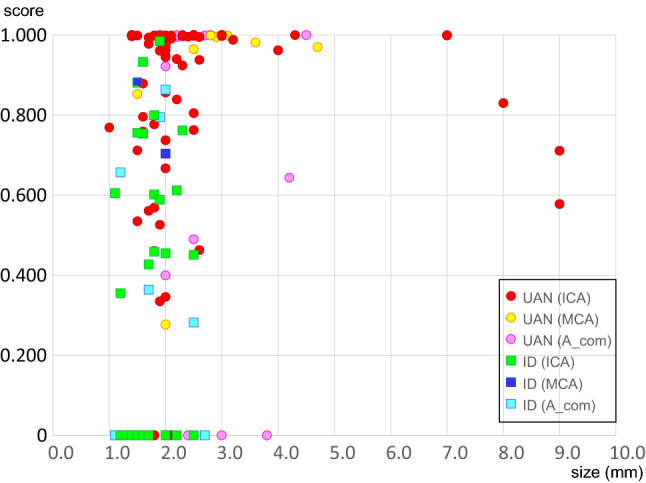
Relationship between the sizes and the scores of the lesions in unruptured aneurysm and infundibular dilatation. Red circle, unruptured aneurysm (UAN) in internal carotid artery (ICA); yellow circle, UAN in middle cerebral artery (MCA); pink circle, UAN in anterior communicating artery (A-com); green square, infundibular dilation (ID) in ICA; dark blue square, ID in MCA; light blue square, ID in A-com. Scores of false-negative lesions are represented as 0.000

Figure 2 shows the relationship between the scores and the lesion size. In either lesion category of the three vascular areas, no significant correlation was noted between the score and the lesion size.

**Fig. 3:**
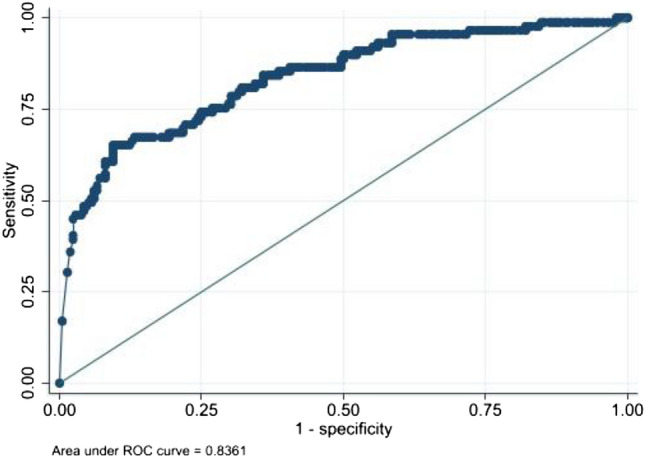
ROC curve of the scores to differentiate UAN from ID or other false positive lesions. *ROC* receiver operating characteristic, *UAN* unruptured aneurysm, *ID* infundibular dilatation

**Fig. 4:**
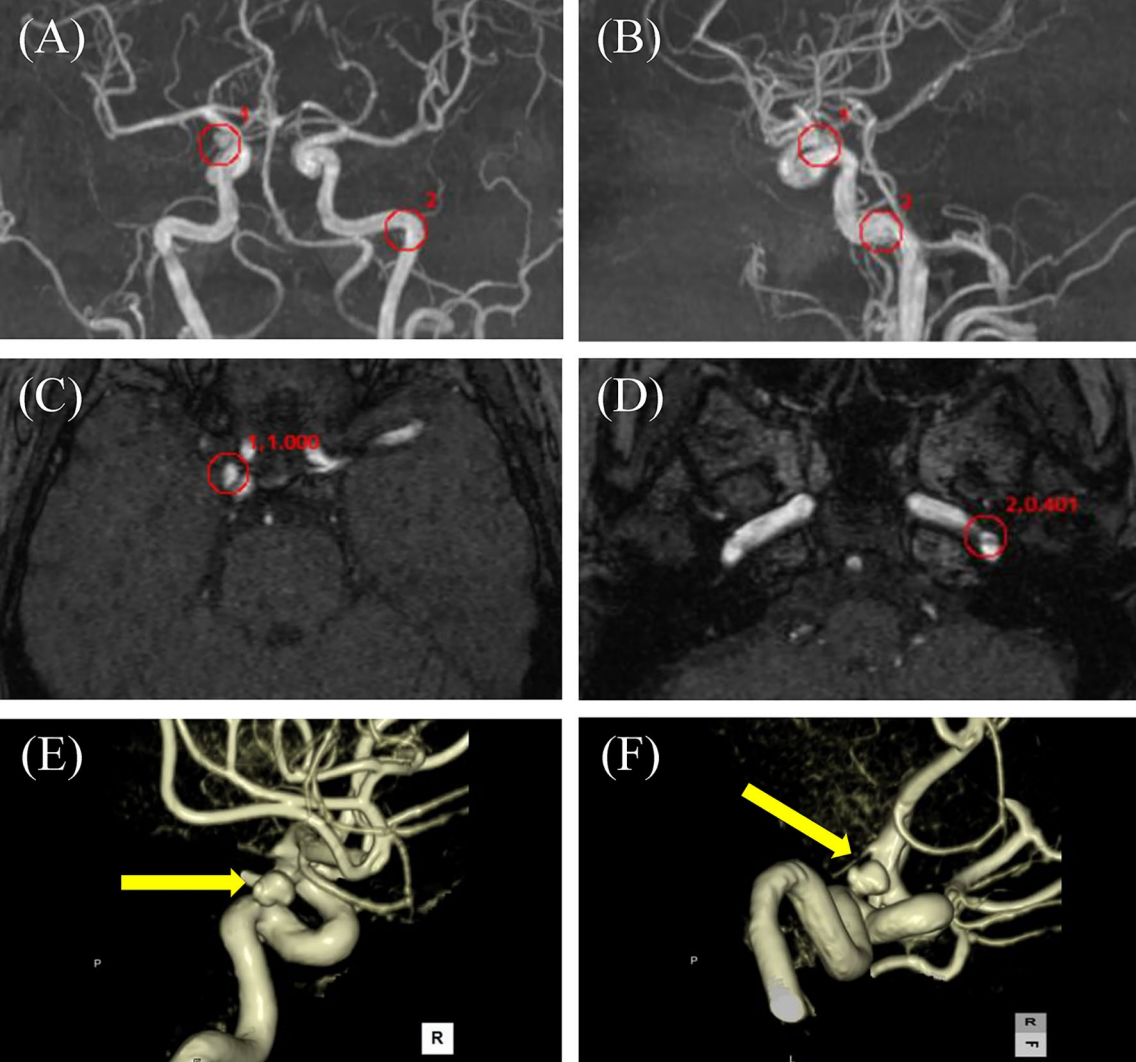
60 s female with single unruptured aneurysm. EIRL brain aneurysm^®^ detected a 4.5 mm (7 mm in angiography) unruptured aneurysm (UAN) in the right internal carotid artery (ICA) and a false positive candidate in the proximal portion of left ICA. Red circles indicate the locations of the candidates, and numbers beside red circles indicate the order and the CNN’s final layer score of the candidate. **A** Frontal MIP image, **B** Lateral MIP image, **C** MRA original image indicating right ICA UAN (1st candidate, score 1.000), **D** MRA original image with a false positive candidate (2nd candidate, score 0.401), **E**, **F** Oblique volume rendering images of 3D-CT angiography (yellow arrow; right ICA UAN). Note that “blebs” are obviously seen in volume rendering images. *CNN* convolution neural network, *MIP* maximal intensity projection, *MRA* magnetic resonance angiography, *CT* computed tomography

**Fig. 5:**
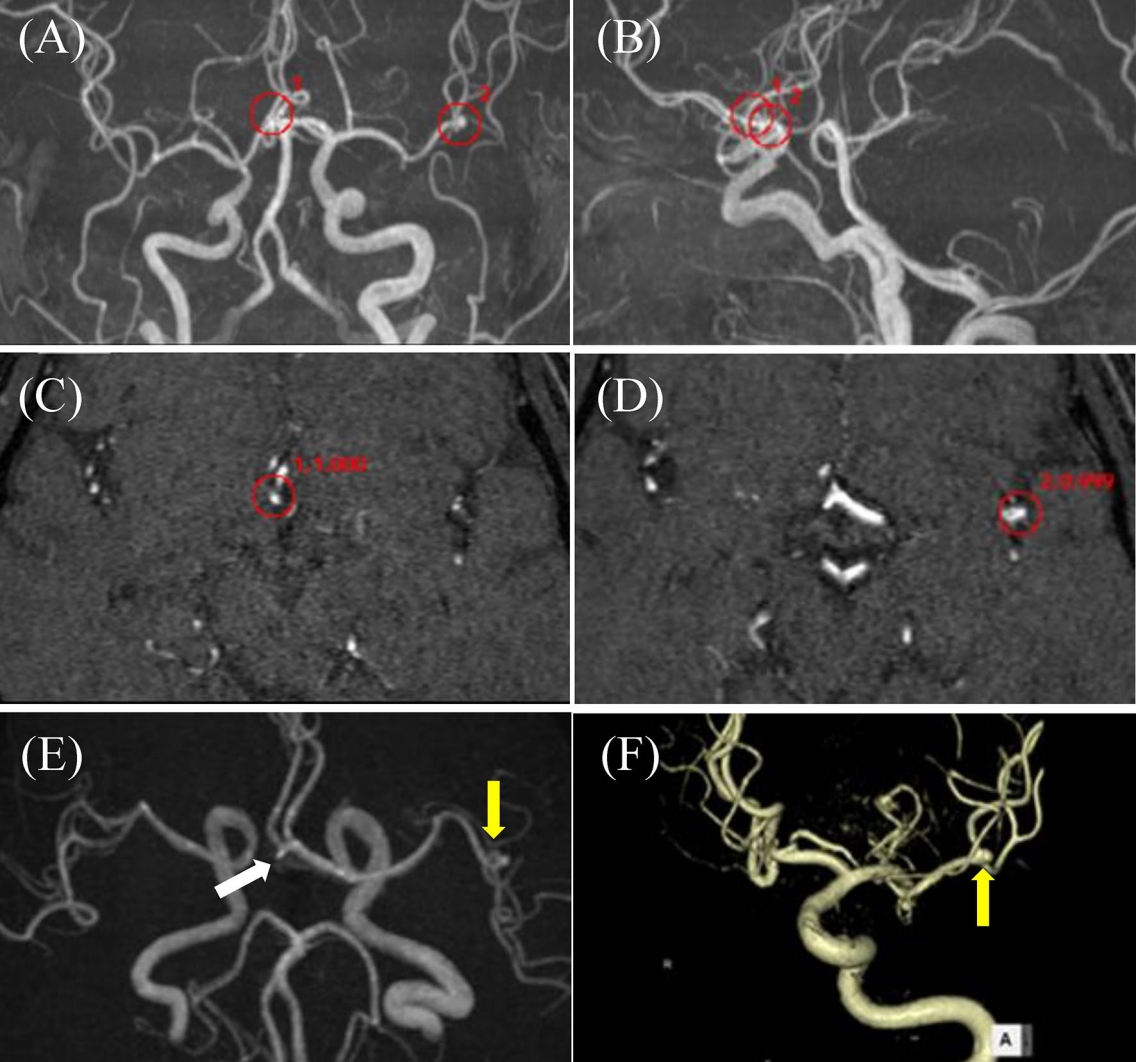
70 s male with double unruptured aneurysms. EIRL brain aneurysm^®^ detected a 2.8 mm unruptured aneurysm (UAN) in anterior communicating artery (A-com) and 3.1 mm UAN in left middle cerebral artery (MCA). Red circles indicate the locations of the candidates, and numbers beside red circles indicate the order and the CNN’s final layer score of the candidate. **A** Front MIP image, **B** Lateral MIP image, **C** MRA original image indicating A-com UAN (1st candidate, score 1.000), **D** MRA original image indicating left MCA UAN (2nd candidate, score 0.999), **E** Frontal MIP image demonstrating double UAN lesions (white and yellow arrows), **F** Oblique volume rendering image of 3D-CT angiography demonstrating left MCA UAN (yellow arrow). *CNN* convolution neural network, *MIP* maximal intensity projection, *MRA* magnetic resonance angiography, *CT* computed tomography

**Fig. 6:**
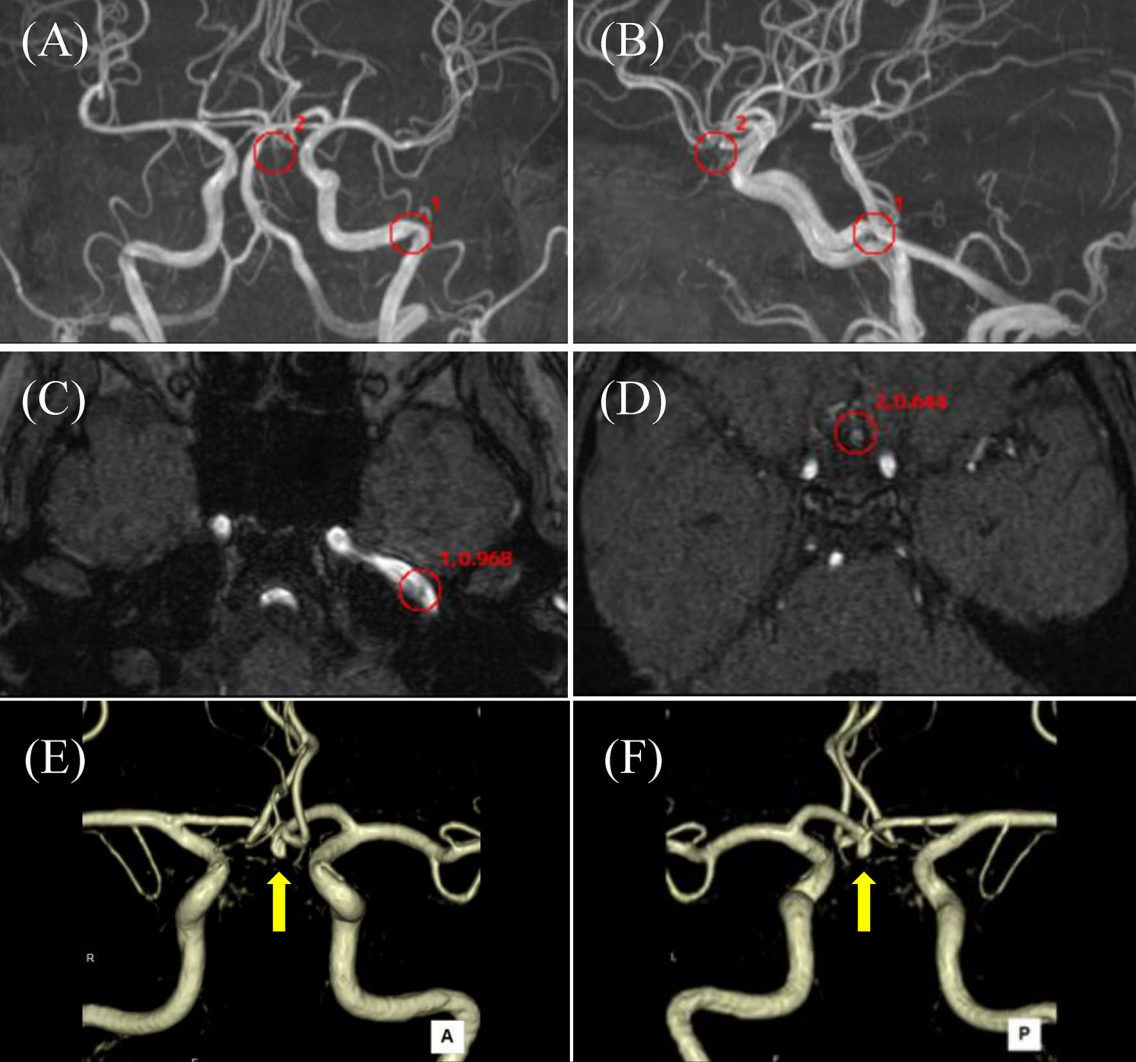
50 s male with single unruptured aneurysm. EIRL brain aneurysm^®^ detected a 4.2 mm unruptured aneurysm (UAN) in anterior communicating artery (A-com) and a false positive candidate in the proximal portion of left internal carotid artery (ICA). Red circles indicate the locations of the candidates, and numbers beside red circles indicate the order and the CNN’s final layer score of the candidate. **A** Front MIP image, **B** Lateral MIP image, **C** MRA original image with a false positive candidate (1st candidate, score 0.968), **D** MRA original image indicating A-com aneurysm (2nd candidate, score 0.644). Note that the signals of A-com UAN are lower than those of the surrounding vessel, **E**, **F** Frontal and inverted frontal volume rendering images of 3D-CT angiography (yellow arrow; A-com UAN). *CNN* convolution neural network, *MIP* maximal intensity projection, *MRA* magnetic resonance angiography, *CT* computed tomography

**Fig. 7:**
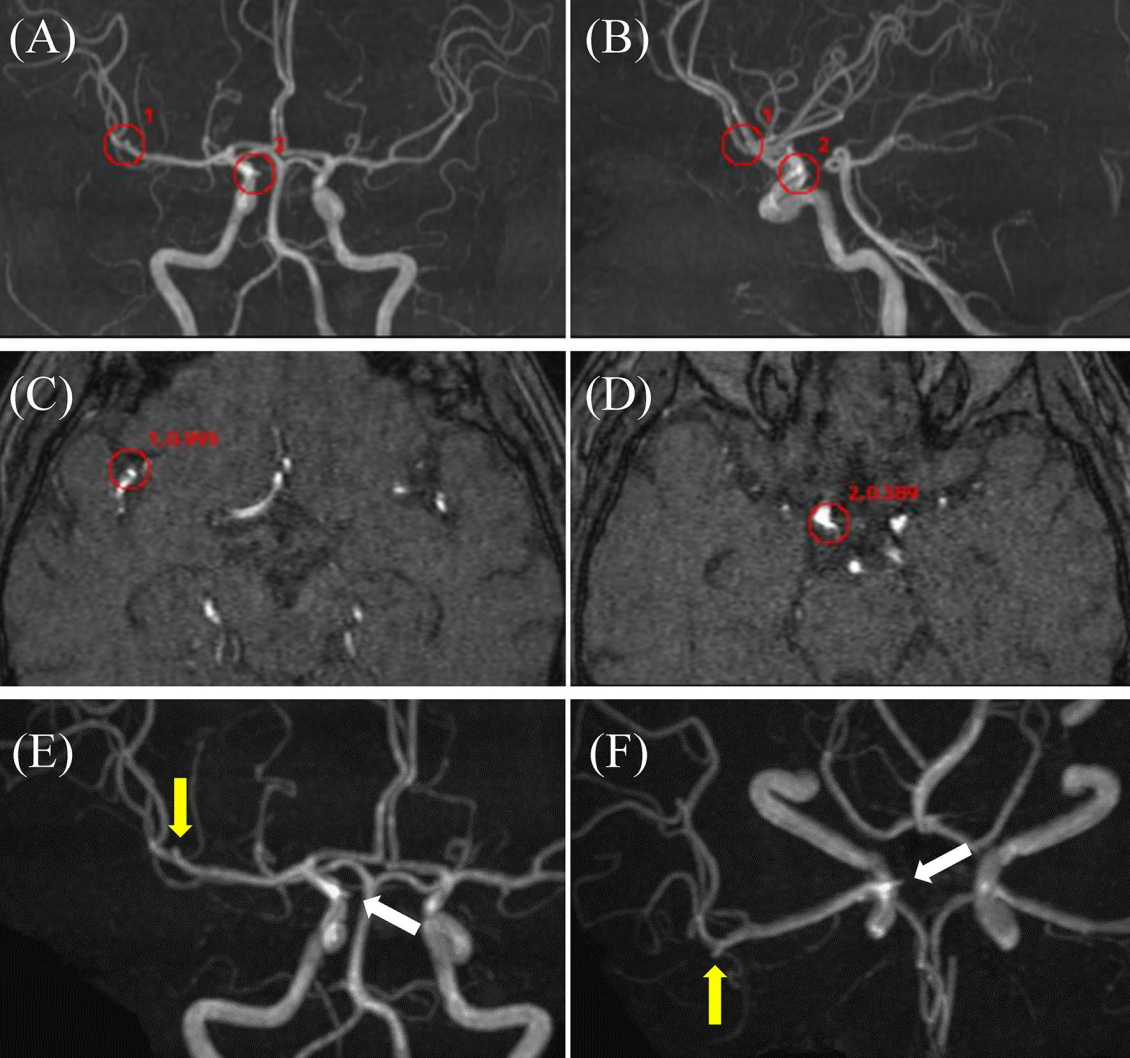
60 s female with single unruptured aneurysm and infundibular dilatation. EIRL brain aneurysm® detected a 2.9 mm unruptured aneurysm (UAN) in right middle cerebral artery (MCA) and a 1.9 mm infundibular dilatation (ID) in right internal carotid artery (ICA). Red circles indicate the locations of the candidates, and numbers beside red circles indicate the order and the CNN’s final layer score of the candidate. **A** Frontal MIP image, **B** Lateral MIP image, **C** MRA original image indicating right MCA UAN (1st candidate, score 0.995), **D** MRA original image indicating right ICA ID (2nd candidate, score 0.589), **E**, **F** Oblique and inverted oblique MIP images of MRA (yellow arrow; rt MCA UAN, white arrow; rt ICA ID). *CNN* convolution neural network, *MIP* maximal intensity projection, *MRA* magnetic resonance angiography

**Fig. 8:**
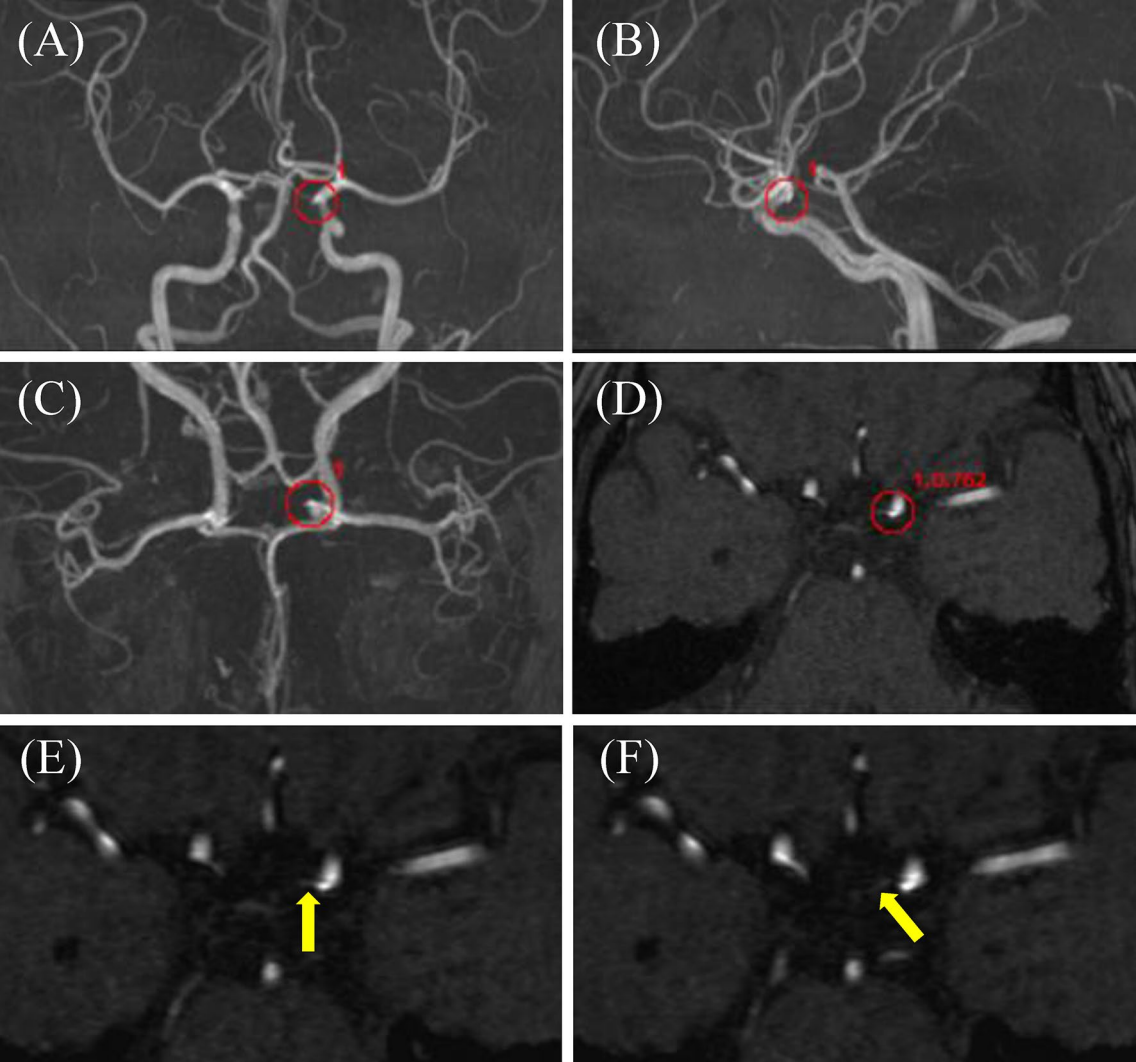
50 s female with single infundibular dilatation. EIRL brain aneurysm® detected a 2.3 mm infundibular dilatation (ID) in left internal carotid artery (ICA). Red circles indicate the locations of the candidates, and numbers beside red circles indicate the order and the CNN’s final layer score of the candidate. **A** Frontal MIP image, **B** Lateral MIP image, **C** Reversed frontal MIP image, **D** MRA original image indicating left ICA ID (1st candidate, score 0.762), **E**, **F** MRA original image demonstrating the tip of ID toward the left posterior communicating artery (yellow arrow). *CNN* convolution neural network, *MIP* maximal intensity projection, *MRA* magnetic resonance angiography

Table 4 shows the summary of the scores of UAN, ID, and normal (false positive) scores in a linear regression model. The score significantly increased to 0.233 (*p* < 0.0001, confidence interval 0.168–0.298) in the rank of candidate 1 and to 0.163 (*p* < 0.05 confidence interval 0.039–0.287) in the rank of candidate 2 in UAN, whereas not in ID when using the normal scores as a baseline. ROC analysis using the score for diagnosing UAN showed a favorable area under the curve (AUC) of 0.836 (Fig. 3). Examples of representative UANs and IDs analyzed by EIRL_BA are shown in Figs. 4, 5, 6, 7, 8.

**Table 4:** The summary of the scores in a linear regression model

	Data set	Coefficient	[95% confidence interval]		*P*
Candidate 1	UAN	0.233	0.168	0.298	0.000
	ID	0.038	− 0.084	0.162	0.533
Candidate 2	UAN	0.163	0.039	0.287	0.010
	ID	− 0.026	− 0.220	0.167	0.787
Candidate 3, 4	UAN	0.153	− 0.073	0.379	0.179
	ID	0.141	− 0.037	0.320	0.118

*UAN* unruptured aneurysm, *ID* infundibular dilatation

## Discussion

In this study, we evaluated the diagnostic performance of CAD considering EIRL_BA as a single reader by comparing it with the diagnosis by the two expert radiologists, which was considered to be the gold standard. A common method of evaluating the diagnostic performance of CAD is to compare the reading accuracy made by radiologists when CAD is used and when it is not used. However, the calculation process of deep learning-based CAD is in a black box, so it is impossible to accurately know the “rationale for how CAD presented the candidates.” Therefore, even if CAD use increases the rate of correct diagnosis by physicians, it will not lead to optimal use if physicians do not know the rationale on which the candidate points were presented. Because optimal use of CAD requires the rationale of CAD detecting the candidates, we independently evaluated the diagnostic performance of the algorithm and examined whether the calculated scores of the CNN’s final layer could be used as the indication for the candidates.

First, the sensitivity for detecting UAN of EIRL_BA was as good as that in the previous reports of deep learning-based CAD systems. Our study also found EIRL-BA useful in detecting relatively small lesions without strictly distinguishing UAN and ID. In fact, the sensitivity of EIRL_BA detecting either UAN or ID was superior to that of single MRA reading for small aneurysms, especially in ICA, by a general radiologist [4]. This is important because both types of lesions should be pointed out in the brain dock, considering that ID could grow rounded, bloated, and UAN in the future [7, 8, 21].

Second, EIRL_BA achieved high specificity in every vascular region, resulting in high values of the positive likelihood ratio. Specificity has not been focused on previous algorithms for cerebral aneurysms to avoid missing abnormality; however, low specificity leads to over-diagnosis and unnecessary follow-up studies with expensive costs and subjects’ anxieties. Therefore, we believe EIRL_BA could be a reliable co-reader to give us valuable information about the disease without such inconvenience.

Finally, we investigated the CNN’s final layer scores based on the shape and curvature of the vascular signal in the original MRA images. If the clue is only the ranking of candidates, inexperienced readers may tend to have a bias: a high ranking of candidates suggests a high possibility of UAN, and a high number of candidates suggests the presence of at least one UAN lesion. However, the results of the linear regression analysis using the scores as an outcome showed that the scores significantly increased in UAN but not in ID, suggesting that the score could be useful for distinguishing between UAN and ID or false positive lesions. CNN is a type of artificial intelligence mimicking the human visual cortex and used for character recognition [22, 23]. EIRL_BA, based on CNN, was trained on the morphological characteristics of UAN but was not trained on those of ID. Therefore, it is natural that the ID scores are equal to those of false positive candidates. In routine readings using MRA, prompt decision whether the current vascular enlargement is UAN or ID is sometimes difficult. In this situation, looking at CNN’s final layer score may be useful to interpret, and referencing the score may be a way to make the right decision without being misled by the rank or number of candidates.

Furthermore, the results of the ROC analysis show the usability of the CNN’s final layer scores as a confidence factor of CAD, which suggests that the score can be used as a reasonable parameter for deep learning-based CAD. EIRL_BA may be helpful for a speedy, concurrent physicians’ reading if images can be read while looking at the score [24]. It is also important that 11% (11/100) of UANs did not enter the candidate. Further study is needed to evaluate whether or not the score contributes to increasing diagnostic accuracy when a physician reads brain MRA alone.

One of the limitations in this study is that the gold standard diagnosis is based on MRA alone. There may be a true UAN or ID lesion among the candidates considered false positive based on the diagnosis by the two radiologists despite correct detection by EIRL-BA. That needs to be verified in a future study. Also, as shown in case 6, it is possible that a low vascular signal in the original MIP image would result in a low score as well. Hence, further validation of false-negative lesions not detected by EIRL_BA is warranted. Additionally, subjects were small sample size from a single institution, especially cases in MCA or A-com were relatively few. Also, this study did not examine the posterior circulation aneurysms, despite it is thought to be one of the important risk factors of rupture [2, 3, 25, 26], which is a subject for further study.

In conclusion, EIRL_BA could be a reliable reader to give physicians valuable information about intracranial aneurysms under a situation where a speedy but accurate reading is required, like “brain dock,” and may be helpful for concurrent reading if the physician can read images while looking at the score; however, further study is needed.
